# Lecture Introductory

**Published:** 1873-11

**Authors:** Robert Battey

**Affiliations:** Professor of Obstetrics


### LECTURE INTRODUCTORY

### TO THE REGULAR COURSE OF LECTURES IN ATLANTA MEDICAL COLLEGE, FOR THE SESSION OF 1873-74.

DELIVERED IN THE HALL OF THE HOUSE OF REPRESENTATIVES NOVEMBER 3, 1873.

BY ROBERT BATTEY, M.D., PROFESSOR OF OBSTETRICS.

Life is real—life is earnest. Useful life is but a succession of days, months, and years of arduous toil.

The Faculty of the Atlanta Medical College has assigned to me the agreeable duty of welcoming you, young gentlemen of the medical class, to the college halls, and to the city of Atlanta.

In the discharge of this duty it would be easy, as it would be pleasant, to enchant your ears with a siren's song. It would be easy to discourse to you of the varied attractions of this beautiful city; of its architectural ornaments, of its attractive surroundings, of its pleasant drives, of its bubbling fountains of health-giving waters, of its refined society, its charming women, its fascinating belles.

I might easily conduct you to the halls of legislation, to our princely mansions, to the opera-house, to the theatre, to the ball-room.

It would be pleasant to assure you that the hill of science is not high; that it is neither steep nor rugged; that the pathway is broad and smoothly paved; that the ascent is handsomely terraced and in easy stages, with convenient and comfortable sittings for rest and recreation, where you might bask yourselves in the sunshine of indulgence, enjoying the lovely tints and fragrant odors of rarest flowers.

Fidelity to truth, however, demands a different course. The consciousness of inflexible duty requires that I should picture to your minds, in the very outset of your professional career, life in its true colors—as a warfare; a perpetual strife; an incessant labor.

From the day when it was decreed of the primeval man, "In the sweat of thy face shalt thou eat bread, till thou return unto the ground," the great law of labor and reward has bound inflexibly the human race, and, as sure as God liveth in the heavens, will bind until time shall be no more.

My colleague, the Professor of Chemistry, will tell you that matter is wholly indestructible. He will tell you that the elements of the coal burning upon your grate are not destroyed at all, as they seem to be, in the combustion, but are only re-arranged in their chemical relations by combination with the oxygen of the air, whereby the carbo-hydrogen compound, coal, is transformed into carbonic acid gas and vapor of water. He will tell you that there is not a single atom of carbon or hydrogen either lost or gained in the transformation, and he will tell you, too, that another of the results of the combustion of coal is the development of force—yes, even of the wonderful force which sends the ponderous railway train thundering along its iron-bound way.

The Professor of Physiology will tell you, doubtless, that every movement of muscle, however slight, even though it be but the winking of the eye-lid or the quivering of the lip, is accompanied by a breaking down of muscular structure and re-arrangement of chemical elements. He will tell you, too, that the merest idle thought, which may flit through your mind, involves disintegration of a certain modicum of brain substance, the chemical elements of which become re-arranged into phosphoric acid, ammonia, carbonic acid, water, etc.

He will tell you that a necessary result of all these chemical changes in organized structures is likewise the elimination of force. He will tell you that the breaking down of muscular and brain tissue is but another form of combustion. He will tell you that deflagrating brain and deflagrating muscle are as indestructible as deflagrating carbo-hydrogen; and let me tell you, too, that the generated force is as enduring, as indestructible as is matter.

Not a movement of your finger, not a thought of your brain is barren of its results. How then, in any true sense, can man be either physically or mentally idle? So long as man breathes, so long as blood continues to circulate through his vascular system; so long as he moves a muscle; so long as his brain evolves a thought, chemical reactions are continually going on within the organism, and continually evolving force. As soon as his system ceases the evolution of force, he returns to the dust from whence he came.

If we may accept the evolution of force as a synonym of labor, then every man is and must be, in an enlarged sense, a continual laborer, and when he ceases to be a laborer, he is dead, irrevocably dead.

The term labor, even in a more restricted sense, cannot, of course, be confined to the mere exercise of muscular power. It includes manifestly, the far loftier and nobler workings of man's more etherial part, the mind. Indeed, mental toil, whether we view it in its enervating and exhausting effects upon the human system, or in the value and magnitude of its results, is *par excellence* the great labor of human life. It is the capacity for mental labor which chiefly distinguishes man from the brute creation.

Man was evidently created for a purpose. When Paley's imaginary traveler picked up from the barren sands of an uninhabited sea-shore, a watch, and viewed its curious fashioning and its intricate machinery, he inferred at once a purpose in its construction; and when man, in the exercise of the reason which God has given him, looks within himself and considers how fearfully and wonderfully he is fashioned, he cannot, for a moment doubt a divine purpose in his creation.

Man owes certain duties to the God who made him. It is the peculiar province of the clergy to enlighten you upon these duties and to exhort you to their faithful performance, and I shall not trench upon the prerogative of their cloth.

But what is more to our present purpose, the individual man owes duties to society in general. By the necessity of our nature as well as by the ordinances of conventionalism, we are mutually dependent creatures. Truly, no man liveth unto himself, no man dieth unto himself. For each and every one there is an allotted task, and the welfare of the whole depends upon the integrity of each individual in the faithful performance of his part.

In the human hive there is no room for drones—each and every man is a worker.

In a narrower, but not less important sphere, man is beset with duties upon every side—as a citizen, as a neighbor, as a friend, as a husband, as a father, as a son, as a brother, as a rational being, as a progressive being, as an immortal being.

Weighed down by such a multiplicity of duties and responsibilities, the life of man is nought but a life of labor.

Nor is man the only laborer. The Omnipotent God who made the heavens and the earth, the sea and all that in them is—the God who rules the destinies of the vast illimitable universe ; the God who holds countless worlds in the hollow of His hand ; the God who rides upon the whirlwind and directs the storm ; the God who orders and directs equally the revolution of a planet and the beating of a pulse, without whose notice not even a sparrow can fall to the ground, is a God of infinite industry and labor.

If we direct our minds to the study of nature and of nature's laws, we find everywhere evidences of purpose in the creation of every living thing that breathes upon the face of the earth and that lives in the great deep ; and we find each and every living creature toiling and striving, day by day, to fulfill the ends of its creation. So too, in the vegetable world, we see everywhere the same evidences of strife to overcome obstacles and to fulfill to the letter the grand objects for which it was made.

Dead mineral matter too, is only dead in its seeming. God has appointed to the rocks even a great and an indispensable work in the economy of nature, and most industriously and faithfully do they perform it. The gentle pattering rain-drops fall upon a rocky ledge, and by insensible degrees fret and wear away its fixed and solid substance. The moisture sinking into its crevices chills and crystalizes under the wintry blast, and by its law of expansion wedges off with irresistable force the surface portions to be further acted upon and disintegrated by the falling rain. It is thus a primitive soil is formed. The spores of minute lichens are now deposited from the hand of the Creator; they germinate, they grow, they mature, they die and decay, adding a small modicum of vegetable humus to the primitive rocky soil, fitting it for the reception of spores from a higher order of lichens, which by their growth and decay in turn prepare the soil for the sustenance of mosses; and so on, in regular progression, until at last the giant of the forest finds deep and firm anchorage for its gnarled roots, and lifts its lofty head towards the heavens and proudly bids defiance to the storm.

Nature abhors a vacuum; the God of nature cannot tolerate in the universe of His creation any useless thing.

Upon you, young gentlemen, who come up here to enter upon a course of study in Medical Science, there rests peculiar and weighty obligations for the earnest and faithful improvement of every hour.

The Science of Medicine, which it is your business to acquire, is nothing but the aggregation of the recorded experience and labor of individual students of medicine in every age and in every clime. It is a great fountain of knowledge to which you are invited, that you may slake your thirst at will. It is freely open to all men who are bidden to come and drink without money and without price.

Medical knowledge is a great common fund, from which the master giveth to each and every one according to his capacity, that he may diligently use the talents committed to his charge, and return the same with usury at his coming. Mark you well: whoever receiveth of this common bounty entereth into an implied but none the less solemn and binding obligation, so to use it that he may in his turn add his own mite to the common wealth.

When you consider that the chart which you seek as evidence of your investment with the dignities of the doctorate, puts into your keeping the weighty responsibility of precious human lives, how well might you tremble with fear of your own insufficiency; how diligent ought you to be in your pupilage, that you may not be weighed in the balance of public opinion and found wanting.

The labor of life has its rewards.

God has implanted in man an inward monitor, whose promptings direct him in the line of duty; an ever present friend, wise and impartial in its judgments, frank and fearless in its admonitions; a friend whose still small voice now speaks words of comfort and of cheer to the feint heart, and now commands the troubled spirit peace, be still! Now it chides and pricks the erring, and now lashes in madness and fury the corrupt.

Under its approving smile virtue becomes its own reward; the bread of the laboring man becomes sweet to his taste, and his rest quiet and refreshing. Writhing under its frown, the unfaithful is ill at ease, sleep departs from his eye-lids and rest from his pillow.

The faithful laborer enjoys the respect and esteem of the world. Those whose good opinion is worth striving for, always hold industry in a high rank among human virtues, and even those whose foolish pride leads them to put on the airs of shabby gentility, and who affect to look down upon honest toil, if they be gifted with but a spark of mother-wit must feel in their hearts deference and true respect for the sturdy hands, soiled though they be, which push onward the car of human progress.

Labor achieves wealth, honors and distinction in the world. The illustrations of this proposition are familiar to you all as household words.

In this land of republican equality there is no favored few, no aristocracy of birth, no hereditary title to wealth, to honor, to fame. All are alike born heirs to the promise. All may alike gratify their laudable ambition. To the possession of these coveted estates there is attached but one condition, and that equally within the reach of all; it is industry, it is application, it is untiring labor.

In an obscure county of Georgia, there lived in years gone by, a youth, born of humble parentage, the offspring of a besotted father. He was inured from early boyhood to arduous toil. He was reared under the blighting influences of the most abject poverty.

At the age of twenty-one he labored upon the streets of his county town, for the slender pittance which purchased a blue-backed Webster's spelling book, and supplied most frugally his bodily necessities, while he availed himself of four or six months instruction in the rudiments of learning at the village school.

A benevolent physician now took him into his employ as a manual laborer, and noting his yearning for knowledge, gave him access to his scanty library. For years he labored hard by day, and trimmed his midnight lamp. In time he sat with me upon the lecture benches, and imbibed the lore of medicine. Now he is in successful practice in a good community, and is noted for his attainments in science. He both reads and speaks two foreign languages, and finds time for researches in natural history.

I knew another young man who had likewise grown up in most abject poverty, and in the blighting atmosphere of the demon whisky. Deprived of all early advantages of education, I marked him well; compelled of necessity to earn his bread by daily toil he improved diligently his hours of rest, storing his mind little by little, and day by day, with useful knowledge. He became an attorney, and now, clothed in wig and ermine, he sits upon the bench, holding in his hand the scales of justice; he commands the respect and confidence of the bar and of the community.

And I knew yet another, for it has been my privilege to note the struggles and to emulate the dilligence and pluck of a goodly number of earnest workers in life.

A young man of twenty-seven, bankrupted in business and burdened with the support of a wife and children, began the study of medicine. By diligent labor and untiring application he has been enabled to avail himself of the instruction of the best medical schools in this country, and likewise of the schools and hospitals of Europe. By continued diligence in business he has accummulated property, and is now in large and lucrative business.

Years ago, in the city of Philadelphia, a boy engaged himself as a servant to one of the Faculty of that venerable Medical School, the University of Pennsylvania. His leisure moments were spent in the Professor's library; his evenings, as chance offered, in the dissecting room, with his crust of bread lieing beside the cadaver; and he munched his scanty meal while he diligently plied the scalpel. For years he has lectured in my Alma-Mater, and now enjoys perhaps the most lucrative surgical practice in America.

But a few months ago the vibrations of the great ocean cable brought to our shores news of the death of Dr. William Tyler Smith, of London.

Descended of humble parentage, he was born in the rural district around the town of Bristol, and, unable, by reason of his poverty, to avail himself of the superior advantages of the metropolitan hospitals, he was educated in the obscure Medical School of his county town.

Very early in his career, with characteristic English pluck, he removed to the great city of London, and had the audacity to beard the great professional lions in their very den.

That old, cosmopolitan Journal of Medicine, the *London Lancet* says of him :

"Tyler Smith, like so many others, who have shed lustre upon their vocation, was in the most absolute sense of the word, a self-made man. Of feeble health, his early education had been necessarily neglected. This circumstance, which to most men would have been an irreparable misfortune, was to him, always self-reliant and ambitious, the spur to the attainment of the noblest and best education for work, that which a strong mind achieves for itself. He entered the Medical School at Bristol, and when it is told that no other door to the temple of Medicine was opened to the poor scholar, the provincial schools have more than justified their existence. The great Metropolitan and University Schools have in our day produced few greater men."

"Tyler Smith began his career as a teacher in the private school of the late Mr. Dermott. The lecture-room was a back kitchen of a house in Bedford Square; the access was by the area steps. But there was a good class, and a teacher whom no hospital in London could surpass. For deficiency in material he made up, by ambition, by a powerful intellect, and by determined industry. His manner was ungainly, his utterance not good, he was not a fluent speaker. In spite of these disadvantages he resolutely declined to write out his lectures, he trusted to spontaneous expression, and by dint of dogged perseverence he became an impressive lecturer and speaker. He was always cool, self-possessed, quick to catch the effect produced by what he was saying. His mind was not sympathetic. He excited little enthusiasm in his hearers. But he rarely failed to instruct, to convince, and to lead. No one could hear him without feeling that he was listening to a powerful intellect, whose working was not merely suggestive, but fruitful in its originality, definite and practical in its conclusions."

"It is given to few men—the one or two only in a generation perhaps—to steer through the rivalry of active professional life in London without giving some ground for criticism. Tyler Smith certainly was not one of these. Nor could he be, starting poor and friendless, with all the world before him, conscious of his own worth, ambitious of fame, and not indifferent to worldly success, he had to struggle hard for both. He could not help seeing, and he felt it keenly, that high place, influence, and profit were too often the heritage of nepotism and the reward of servile mediocrity. He boldly asserted his own superior right; he fought for it; won it by sheer force of character, by unflinching tenacity of purpose, and by the yet higher merit of honest and brilliant work. In the fierce struggle he left some of his competitors wounded on the field, himself not unscathed. These incidents in his personal career will soon be forgotten. They cannot linger even in the breasts of those of his survivors, who at one time felt themselves aggrieved, and this can truly be said of him, which cannot be said of many men, that he has left behind him, not only a competent inheritance for his family, but a great and enduring legacy to mankind, and an honorable name to be added to the long list of the worthies of British medicine."

I was myself personal witness to the initiatory step in Tyler Smith's surgical career. While sojourning briefly in London, in the year 1859—I was invited to address the Obstetrical Society, of which he was the founder, upon the subjet of my recently perfected method of operating vesico-vaginal fistula. In leaving the hall, Tyler Smith took me by the arm and inquired: "How is it, Doctor, that the women of America permit a mere boy, like you, to practice so delicate and difficult an operation?" I explained to him that our country was a land of freedom in fact, as well as in name; that we were not hampered by the prestige of gray-hairs or influential position; that knowledge and skill were never wanting in opportunity.

"Let me tell you," said he, "I have encountered very many of these cases, as you must know, and have always turned them over to the surgeons. To-night, for the first time in my life, the thought has occurred to me that I might do this operation for myself, and I shall try the very next case that offers."

Scarce six years had elapsed when Tyler Smith not only did vesico-vaginal fistula successfully, but had reported to the same Society brilliant achievements of his skillful knife, in the much grander field, ovariotomy.

A few years ago the whole Medical World was clothed in sackcloth, by reason of the sudden and unexpected death of one who sat upon the very pinnacle of professional fame. I allude of course to Sir James Simpson, of Edinboro, who, besides the inestimable boon of chloroform, gave to the world so many valuable discoveries as to stamp his name indellibly upon the memory of man in all coming generations.

Humble and unworthy as I was, it was once my privilege to bear to him an introductory letter, from Professor Fleetwood Churchill, of Dublin; to take him by the hand; to call him my friend. It was my privilege to become, for a brief season, an inmate of his hospitable house; to share the bounty of his board; to learn wisdom from his lips; to accompany him in his rounds of visitation, and to assist him in his operations. Never was there a more impressive example of the golden fruits of industrious labor than was exhibited in the career of Simpson. He was, in the highest sense, a self-made man.

In the zenith of his professional fame, I can myself testify to his gigantic labors. In the morning hour, while he breakfasted, the long line of carriages in front of his Queen street residence was rapidly forming, and patients as rapidly filling up his spacious reception halls. Through the day the tide poured in to fill every vacancy, and as daylight closed, it was the constant duty of the butler to turn away scores who had patiently waited for long hours in vain. The six o'clock dinner over, he spent a few brief moments in the family circle, and took me in his carriage from house to house, rarely completing the round before one and often two in the morning, At the eight o'clock breakfast, his cheerful smiling countenance was always the center of attraction.

Nor was Sir James content with his own herculean labors. He brought into requisition a host of subordinate minds, all guided and directed by his own gigantic intellect, and supplying more or less crudely elaborated thought for his own mental finishing.

To those of you, young gentlemen, who have been raised upon the farm, whose hardened palms have been made familiar with the plough handles—to those who from boyhood have been inured to honest labor; to those who have been early taught "whatsoever thy hand findeth to do, do it with all thy might," I offer my earnest congratulations. Let me assure you, that it is of such material that true men are made. Let me assure you, too, that when once thoroughly trained in this discipline, the battle of life is already half fought.

If there be among you one whose ill fortune it is to have been born with a silver spoon in his mouth; one who has imbibed his infantile pap from a silver porringer; one reared in the lap of luxury and self-indulgence, with softened muscles and weak, irresolute nerves, let me extend to him my heartfelt sympathy and commiseration. Let me admonish him that it is not yet too late, perhaps, to retrieve the past. Though he may not be capable at once of a firm and manly resolution, let him but make the effort, and he will find that his muscles will yet harden by exercise, his nerves gain strength, and his brain acquire vigor by exertion.

Young gentlemen, we bid you again a hearty welcome. We pledge you, upon our part, earnest and untiring efforts well and truly to expound to you the great truths of Medical Science, and every diligence to successfully induct you into the Arts of Medicine in all its branches. And we exhort you to acquit yourselves as men. Be resolute workers, both in the class-room and in your study. Be true men. Be strong men.

Ladies and Gentlemen of Atlanta: A few months ago, while quietly pursuing my vocation as an humble disciple of Æsculapius in the city of the mountains, an unexpected summons reached my cottage-home on the bank of the Etowah, from the Trustees and Faculty of the Atlanta Medical College, saying, "We have work to do, come thou and help us."

At the break of morn, over hill and dale, resounds the shrill cry of the pack in hot pursuit of the cunning fox; it strikes the eager ear of the hunter and his heart leaps with a thrill of enthusiasm as he plunges the rowel into his steed, and regardless of obstacles, dashes onward in the headlong chase. So, to me, comes the clarion sound, "there is work—there is work to do!"

In obedience to this call, and at the instance of those whom I represent, I appear before you to-night bearing a message.

Shall I tell you that the fame of your great and growing city, the energy, the enterprise, the public spirit of your citizens, are not unknown to me. Shall I tell you how, long years ago when Atlanta was but just in the bud, as it were, when the old Atlanta Hotel, now gone and nearly forgotten, was bran span new, I spent a night under its roof; how I eyed, with suspicion, the rough, hardy band of adventurers who thronged its halls; how my boyish hand instinctively clutched my watch and slender purse beneath my pillow while I slept. Shall I tell you how, as you added house to house, store to store, shop to shop, I lent a willing ear to the croakers and thought surely this can't continue long. Shall I tell you that when you began to wax strong and spread out your arms to embrace new territory, I thought to myself this great bubble needs but the prick of a pin and straightway it will collapse. Shall I tell you that when the hand of the hostile invader swept your fair city from the face of the ground, as I viewed in sorrow its blackened ruins, I imagined no one would be so rash as to build it again.

Shall I tell you that the logic of events has forced me at last to accept the great city of Atlanta as a *stubborn fact.* Shall I tell you that I have come to you with an open heart and a willing hand, that you may teach me the magic of your effective work.

I might tell you all this and much more, but it would be foreign to my present purpose. I address you as the mouth-piece of the College-Faculty, and I am commissioned to say that we have work for you to do.

It is the purpose of those who have this institution in especial charge to make the present winter an epoch in its history. Already enjoying a liberal patronage, it is our purpose to re-double our diligence, to add more and more to its educational advantages, to attract larger and larger classes to its halls.

Already, we learn, that a considerable fund has been accu-mulated, largely through the self-sacrificing efforts of a fair daughter, the Florence Nightingale of Atlanta, for the building of a City Hospital. In this enterprise we feel the deepest inter-est, and to it we shall give our hearty support.

We purpose, likewise, to encourage and aid the establish-ment of projected hospitals for the treatment of special diseases at this point. We desire, in a word, that Atlanta shall be made, what she is entitled by her geographical position to become, the great medical centre of the South. We desire that facilities shall be centered here for the skillful treatment of intricate dis-ease in all its forms ; that the afflicted may come from the four winds of the South to be healed. We ask your hearty co-opera-tion and aid in these enterprises.

For you, young maidens, we have work to do. In the records of ancient history, both sacred and profane, we find nu-merous examples of the marvelous power of woman to wield the destinies of the strongest men. Indeed, in the story of Adam and Eve, of Sampson and Delilah, of Marc Antony and Cleo-patra, we read the history of woman's control in every age, in every land, and in every station in society.

The stout heart which rides sternly over the obstacles of life; which bears the warrior proudly on to triumphant victory; which faces unmoved the terrors of the ocean storm; which endures alike the fierce heat of the tropics and the benumbing chill of the polar seas; yields itself an easy prey to the gentle, winning voice of woman. In her soft and delicate hand, man's hard and flinty heart melts and moulds like plastic potters' clay.

The influence of this magical power we see around us upon every hand; day by day we feel its hidden but resistless impulse deep down in the recesses of our own hearts.

Need I remind you how easily the fascinating charms which attach to your sex and youthful bloom may draw away the minds and hearts of these young men from the labor before them into the more inviting fields of romance and sentiment.

Need I implore you so to use your potent influence as to fire their young hearts with laudable pride and ambition to excell in their studies; to make dilligent use of their opportunities; to faithfully improve every moment of time.

Do this, and in due time we will return to you men; strong men; men upon whose arms you may safely lean in trusting confidence; men to whose softly whispered vows you may venture to lend a listening ear; true men, whose loving smiles may well stir in your responsive hearts not the flutter of reciprocal affection only, but the upheavings of conscious pride.

For you, stately and respected matrons of Atlanta, we have work to do. By the winning force of a mother's smile and a mother's sympathy, stimulate these young men too, in zeal and energy. Warn them against the temptations and allurements of city life which beset them on every side. Invite them into the paths of usefulness, of dilligence, of sobriety, of virtue. Should the hand of disease lay heavily upon one of them, and he be forced to relinquish his work, with the soft, gentle hand of woman, smooth the pillow of suffering, cool the fevered brow, moisten the parched tongue. When human help faileth, with woman's trusting faith, point him to the higher source of hope. When the breath of life ceases and returns to the God who breathed it forth, remember that somebody's darling child is dead; drop a woman's tears of sympathy over his bier and imprint upon the cold, marble brow the parting kiss of the broken-hearted mother at home.

For you, strong men of Atlanta, we have work to do. We commend to your kindly interest the institution in which we labor; visit it, inform yourselves of its practical workings, and you can do much to sustain it in the estimation of the public; to increase its classes and extend its usefulness.

Push forward with your influence and material contributions the City Hospital, and the special hospitals which are projected and will soon be in operation in your midst.

For you, gentlemen of the Board of Trustees, we have work to do. Watch vigilantly over the institution committed to your keeping; guard it carefully against foes from without and foes within. In as far as you may find us earnestly and faithfully laboring for its advancement, give us your hearty co-operation and encourgement. If at any time, you should find us wanting in diligence and capacity for our work, we would have you displace us at once, and put worthier laborers into the field.

In conclusion, let me remind you, my brethren of the Faculty, that the burden and heat of the day's work devolves upon us. When we consider that the young men educated here are destined to go out into the world, bearing the diploma of the Atlanta Medical College as a certificate of their professional proficiency, and become charged with the health and the lives of their fellow-men ; how fearful is the responsibility which will rest upon us.

The people of the Commonwealth of Georgia, in chartering this institution, have imposed upon us great and weighty obligations, which demand at our hands the greatest vigilance and most untiring labor.

In fear and trembling at the magnitude of our duty, let us kneel together at the shrine of Medicine, and pledge each to the others, our full devotion to the task.

---

Devotion to Science.—A remarkable instance of devotion to science occurred in the case of Dr. Otto Obermeier, who died in Berlin, August 19th, from cholera. For several months he had been engaged in examinations of blood in typhus fever, and later, in researches on cholera. He was in the habit of keeping in his bed-room specimens taken from patients who died of cholera and also portions of their excreta. When aware of his condition he made several microscopic examinations of his own blood, although death followed in a few hours.—*N. Y. Med. Jour.*

In the *Aertzl. Intell. Bl.* Dr. Welsch suggests the use of iodine in the treatment of croup, and relates a case in which the employment of the drug in daily doses of one drop to a tumblerful of water was extremely successful.

